# Conventional plate fixation versus minimally invasive modified pedicle screw-rod fixation for anterior pelvic ring fractures

**DOI:** 10.1371/journal.pone.0215233

**Published:** 2019-04-08

**Authors:** Yao-Tung Tsai, Chun-Liang Hsu, Chun-Chi Hung, Yu-Ching Chou, Chia-Chun Wu, Tsu-Te Yeh

**Affiliations:** 1 Department of Orthopaedic Surgery, Tri-Service General Hospital and National Defense Medical Center, Taipei City, Taiwan, Republic of China; 2 School of Public Health, National Defense Medical Center, Taipei City, Taiwan, Republic of China; Universidade Federal Fluminense, BRAZIL

## Abstract

There are various methods of fixation for anterior pelvic ring fractures. We report our experience with modified pedicle screw-rod fixation (MPSRF) via a minimally invasive method. Fourteen patients with anterior pelvic ring fracture were treated by internal fixation (conventional plate fixation, n = 7; MPSRF, n = 7). Intraoperative blood loss, operative time, post-operative fracture reduction grading by radiography, and complication rates were compared and statistically analyzed. Patients treated with MPSRF had a shorter operative time (-36 min, P = 0.378) and significantly less blood loss (-264 ml, P = 0.026) than patients in the conventional plate fixation group. Postoperative radiological evaluations were similar between the groups (P > 0.05). The complication rate was lower in the MPSRF group (1/7 patients) than in the plate fixation group (2/7 patients). Modified pedicle screw-rod fixation is a viable treatment for anterior pelvic ring fixation and can reduce blood loss.

## Introduction

The pelvic ring structure is composed of two innominate bones and the sacrum. The anterior pelvic ring consists of the bilateral pubic rami connected by the pubic symphysis. The posterior ring includes the sacrum and two innominate bones joined at the sacroiliac joints by ligaments. The posterior ring provides 70% of pelvic stability and the anterior ring provides the remaining 30% [1]. Bruce et al. showed that posterior ring fracture involving portions of the anterior pelvic ring is more likely to develop displacement [2]. In order to achieve better reduction and stable fixation, combined posterior and anterior fixation is important. Anterior ring fixation is performed for insecure posterior ring fixation, augmentation of the pelvic ring, or isolated straddle fracture. Anterior pelvic ring stabilization can be achieved by external fixation or internal fixation. The internal fixation methods include a plate [3], an antegrade or retrograde screw [4], a subcutaneous pelvic bridge by the occipitocervical spinal implant [5], an anterior pelvic internal fixator with supra-acetabular spinal pedicle screws and a subcutaneous connecting rod (INFIX), or modifications of the INFIX [6–8].

Traditionally, large wound exposure, longer surgical time, increased intraoperative blood loss, and risk of neurovascular injury have been reported as disadvantages of plate fixation [9]. In this study, we used a method in which a submuscular pedicle screw-rod fixation device was placed through a small incision over the iliac wing and above the pubic symphysis region. Compared with the INFIX method, we modified the pedicle screw position from the anterior inferior iliac spine to the upper inner table of the iliac fossa and the superior pubic rami, fixed with a connecting rod. The lumbar pedicle screw used in the INFIX was replaced by a cervical pedicle screw in our modified method. The aim of this study was to assess the clinical results of anterior pelvic fracture treatment using the conventional plate fixation method compared to our modified pedicle-screw rod fixation (MPSRF) method.

## Materials and methods

This was a retrospective case-series study approved by the Institutional Review Board of the Tri-Service General Hospital (Approval no.: 2-106-05-092). Evaluation of medical documents and radiographic images of patients with anterior pelvic ring fracture treated with plate fixation or MPSRF at the Tri-Service General Hospital between June 2011 and May 2018 was conducted. Informed consent was obtained from all patients included in the study.

We included 14 patients who sustained fracture of the anterior pelvic ring and were treated surgically. Inclusion criterion was a diagnosis of pelvic fracture with anterior pelvic ring involvement. Exclusion criteria were age younger than 20 years, a metastatic tumor in the pelvic region, associated brain injury or neurovascular injury, and pubic rami fractures treated with screw fixation only. The patients were divided into two groups: the conventional plate fixation group (group 1, treated between June 2011 and September 2014) and the MPSRF group (group 2, treated between October 2014 and May 2018). Diagnostic radiography, including pelvic anteroposterior (AP), inlet, and outlet views and computed tomography (3-mm axial slices) were performed for detailed pre-operative examination in all patients. Fracture classification was performed according to the Tile classification [10].

### Surgical technique

**Fig 1:**
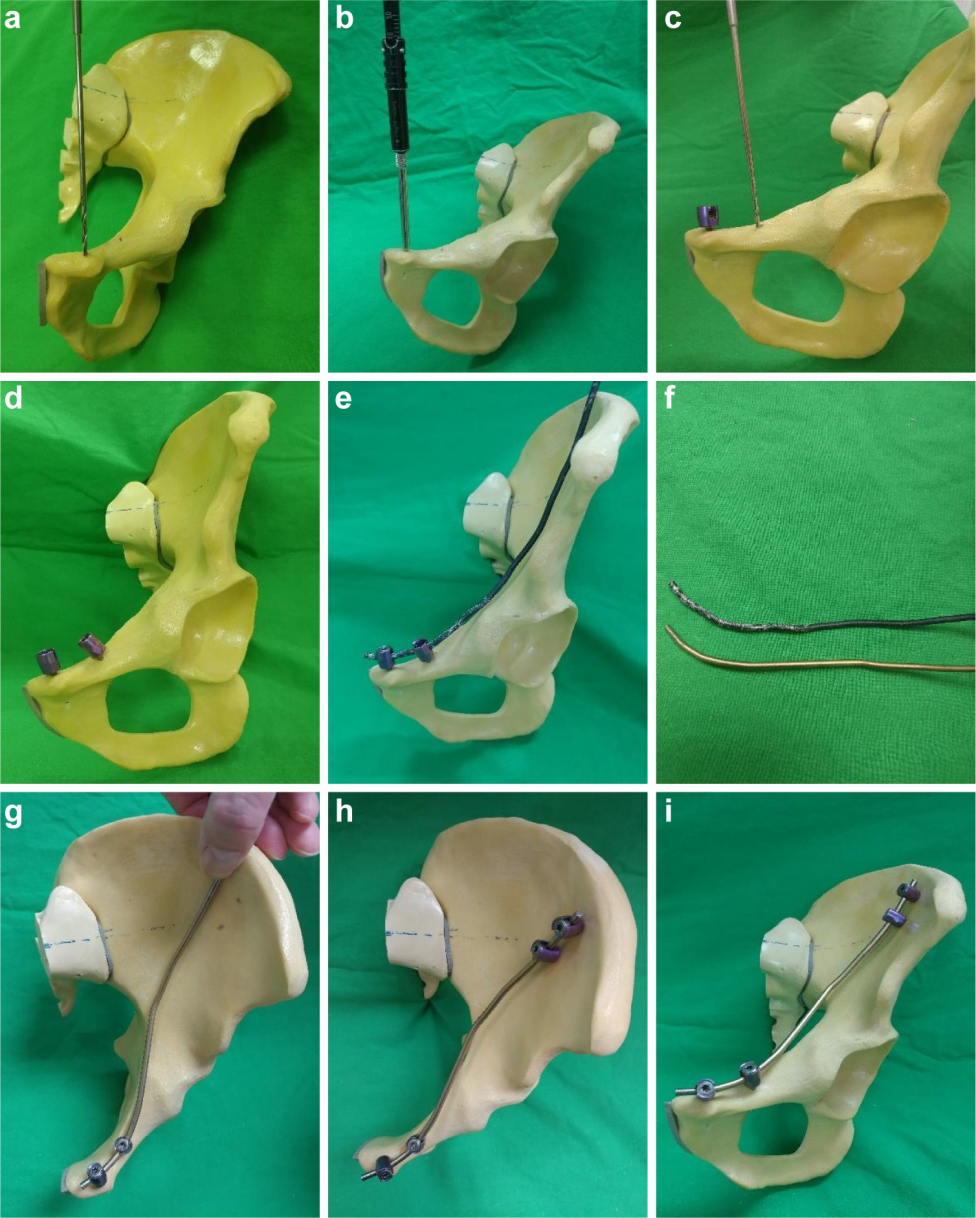
Surgical steps of modified pedicle screw-rod fixation (MPSRF) on a sawbone model. (a) Drilling the pedicle screw hole on the superior pubic ramus. (b) Gauging the screw length and 3.5-mm pedicle screw application. (c) Drilling the second screw hole; in our MPSRF method, two pedicle screws on the superior pubic ramus are needed. (d) Two 3.5-mm polyaxial pedicle screw are inserted. (e) Passing the rod template through the submuscular tunnel; the template is applied on the 2 pedicle screws to adjust the rod figure. (f) Contouring the connecting rod with the rod template. (g) Temporary application of the rod to determine the remaining two pedicle screw positions over the inner table of the iliac wing. (h) Repeat drilling, gauging, and applying the remaining two 4.0-mm pedicle screws and connecting these pedicle screws with the rod. (i) Post-fixation hemi-pelvic anterior-posterior view.

All surgeries were performed by one senior orthopedic pelvic trauma surgeon in our department. Included patients underwent posterior pelvic ring fixation if an unstable posterior pelvic ring was present. Anterior pelvic ring fixation was performed in a separate staged surgery. All patients were placed in the supine position on a radiolucent table. In group 1, the ilioinguinal approach was used. We used a thin metal template to shape the bone contour and estimate length intraoperatively. According to the template, the plate was contoured to conform to the fracture surface of the iliac bone and superior pubic ramus. A commercial in-situ plate bending instrument (Depuy Synthes, Switzerland) was used for minor plate adjustments. The modified Stoppa approach accompanied by a lateral window of the ilioinguinal approach was used in group 2. We used two incisions for unilateral anterior pelvic ring fracture and three incisions for bilateral fractures. The lateral incision was made 1 cm above the anterior superior iliac spine (ASIS) and extended approximately 5–6 cm posteriorly. The medial incision (approximately 5–6 cm in length) was made on the superior pubic symphysis. The tunnel between these two incisions was made by partial elevation of the iliacus muscle on the inner plane of the iliac wing with blunt dissection. A hip flexion maneuver can help with muscle relaxation. Two 3.5-mm-diameter and two 4.0-mm-diameter Axon Spine System (Depuy Synthes, Switzerland) polyaxial pedicle screws were fixed on the superior pubic ramus and approximately 4–5 cm medial to the ASIS, respectively. When screws were fixed on both sides and a temporary reduction was attained, a rod was connected between the pedicle screws through the sub-muscular tunnel. The rod was fixed to the pedicle screw heads with caps locked to maintain the reduction (Fig 1). Immediate fluoroscopic evaluation with pelvic AP, inlet, and outlet views was performed before wound closure.

Rehabilitation began 1 week after the operation and included passive range of motion, quadriceps strengthening, and no weight-bearing for 6 weeks postoperatively. Partial weight-bearing with crutch-assistance was allowed when radiography performed during outpatient follow-up indicated partial callus formation. Full weight-bearing was allowed after 8 weeks.

### Outcome measures

We used the radiographic pelvic series (AP, inlet, and outlet views) for post-operative assessment. Follow-up radiographs demonstrated the fracture reduction quality and plate position. All patients were followed up in the outpatient department at 2 weeks, 4 weeks, 8 weeks, 3 months, 6 months, and 1 year after discharge. All post-operative radiographs were evaluated by 3 trauma surgeons, and analysis of fracture reduction quality was determined by consensus. The result was stratified according to the quality of fracture reduction of the anterior pelvic ring (graded as excellent (0–5 mm displacement), good (5–10 mm displacement), and fair (10–20 mm displacement)). The total operative time, blood loss volume, and quality of fracture reduction were evaluated. The total operative time was defined as the time from skin incision to skin closure and blood loss volume was recorded in the medical record. Specific complications, including injury of visceral organs, the femoral artery, vein, or nerve, the lateral femoral cutaneous nerve (LFCN), and the round ligament (females) or spermatic cord (males) were assessed. Other complications including wound infections, soft tissue impingement by the implant, implant failure, loss of reduction, heterotopic ossification, and non-union were also recorded.

Demographic data are reported as mean ± standard deviation for continuous and categorical variables. Clinical characteristics were compared using the independent *t*-test or chi-squared test and the Fisher’s exact test for categorical variables. All statistical assessments were two-tailed, and P < 0.05 was defined as statistically significant. The Statistical Package for the Social Sciences software (version 22.0, IBM Corporation, Somers, NY, USA) was used for all statistical analyses.

## Results

### Clinical data

Age, sex, mean body mass index, Tile classification, unilateral or bilateral involvement, and implant number are summarized according to group in Table 1. Overall, there was no significant difference in clinical demographic data between the two groups (P > 0.05). The duration of follow-up was 25.3 months (range, 5–67 months). No patient was lost to follow-up.

**Table 1:** Demographic data and clinical characteristics.

	Group 1 (n = 7)	Group 2 (n = 7)	**P value**
**Age, M±SD**	44.71±20.00	36.00±7.33	0.312^a^
**Sex**			0.429^b^
** Male**	4	2	
** Female**	3	5	
**BMI (kg/m**^**2**^**)**	23.57±3.23	22.47±4.83	0.625^a^
**Tile classification**			1.000^b^
** B**	4	5	
** C**	3	2	
**Day (from injury to operation)**	8.00±3.96	6.43±4.47	0.499^a^
**Implant**			1.000^b^
** Bilateral**	3	3	
** Unilateral**	4	4	
**Implant number**			1.000^b^
** 1**	4	4	
** 2**	3	3	

n: number; BMI: body mass index; M±SD: mean ± standard deviation

^a^t test or chi-square test

^b^Fisher’s exact test

In group 1, posterior pelvic ring fixation was performed using iliosacral screw fixation in 3 patients and iliosacral screw combined with spinopelvic fixation in 1 patient. In group 2, iliosacral screw, iliosacral screw combined with spinopelvic fixation, spinopelvic fixation, transiliac plate, and transiliac plate combined with spinopelvic fixation were utilized for posterior pelvic ring fracture in 1, 2, 1, 1, and 1 patient, respectively. A summary of surgical procedures used for all the patients is provided in Table 2.

**Table 2:** A summary of surgical procedures used for all the patients.

Patient	Group	Tile classification	Surgical procedure		Implant side of the anterior ring
			Anterior instrumentation	Posterior instrumentation	
**1**	1	B	Plate	None	B
**2**	1	B	Plate	None	U
**3**	1	C	Plate	Iliosacral screw	U
**4**	1	B	Plate	Iliosacrsal screw	U
**5**	1	C	Plate	Iliosacral screw	B
**6**	1	C	Plate	Iliosacral screw	U
**7**	1	B	Plate	None	B
**8**	2	B	MPSRF	Iliosacral screw + spinopelvic fixation	U
**9**	2	B	MPSRF	Spinopelvic fixation	B
**10**	2	B	MPSRF	Transiliac plate	U
**11**	2	C	MPSRF	Transiliac plate + spinopelvic fixation	B
**12**	2	C	MPSRF	Iliosacral screw + spinopelvic fixation	U
**13**	2	B	MPSRF	None	B
**14**	2	B	MPSRF	Iliosacral screw	U

M male, F female, MPSRF modified pedicle screw-rod fixation, U unilateral, B bilateral

### Perioperative clinical parameters

The total operative time was 280.86 ± 79.68 minutes in group 1 and 245.00 ± 66.39 minutes in group 2 (P = 0.378). The average blood loss was significantly different between the groups (group 1: 601.43 ± 153.78 ml and group 2: 337.14 ± 229.11 ml, P = 0.026).

### Post-operative radiographic results

**Fig 2:**
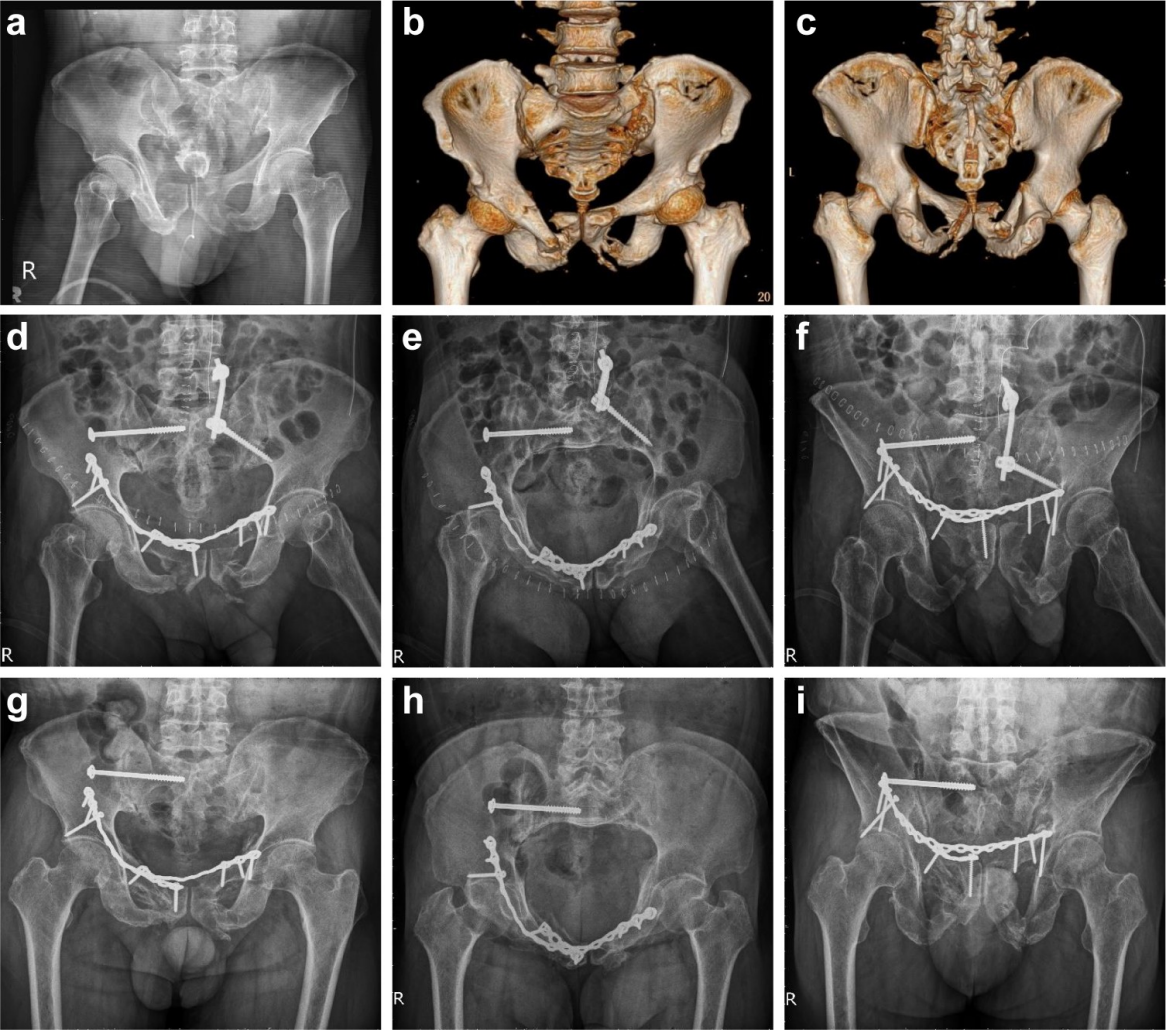
A 62-year-old male with anterior and posterior pelvic ring injury. **a** Preoperative pelvic anterior-posterior (AP) view. **b** Preoperative 3D reconstructed computed tomography (CT) anterior view. **c** Preoperative 3D reconstructed CT posterior view showing sacral fracture with bilateral superior and inferior pubic rami fracture. **d, e, f** Postoperative pelvic radiology series (AP, inlet, outlet view) demonstrating percutaneous iliosacral screw, spinopelvic fixation of the posterior pelvic ring, and conventional plate fixation of the anterior pelvic ring. **g, h, i** Postoperative pelvic radiology series (AP, inlet, outlet view) at 12-month follow-up, demonstrating bone union and removal of spinopelvic fixation. The quality of fracture reduction was graded as good.

**Fig 3:**
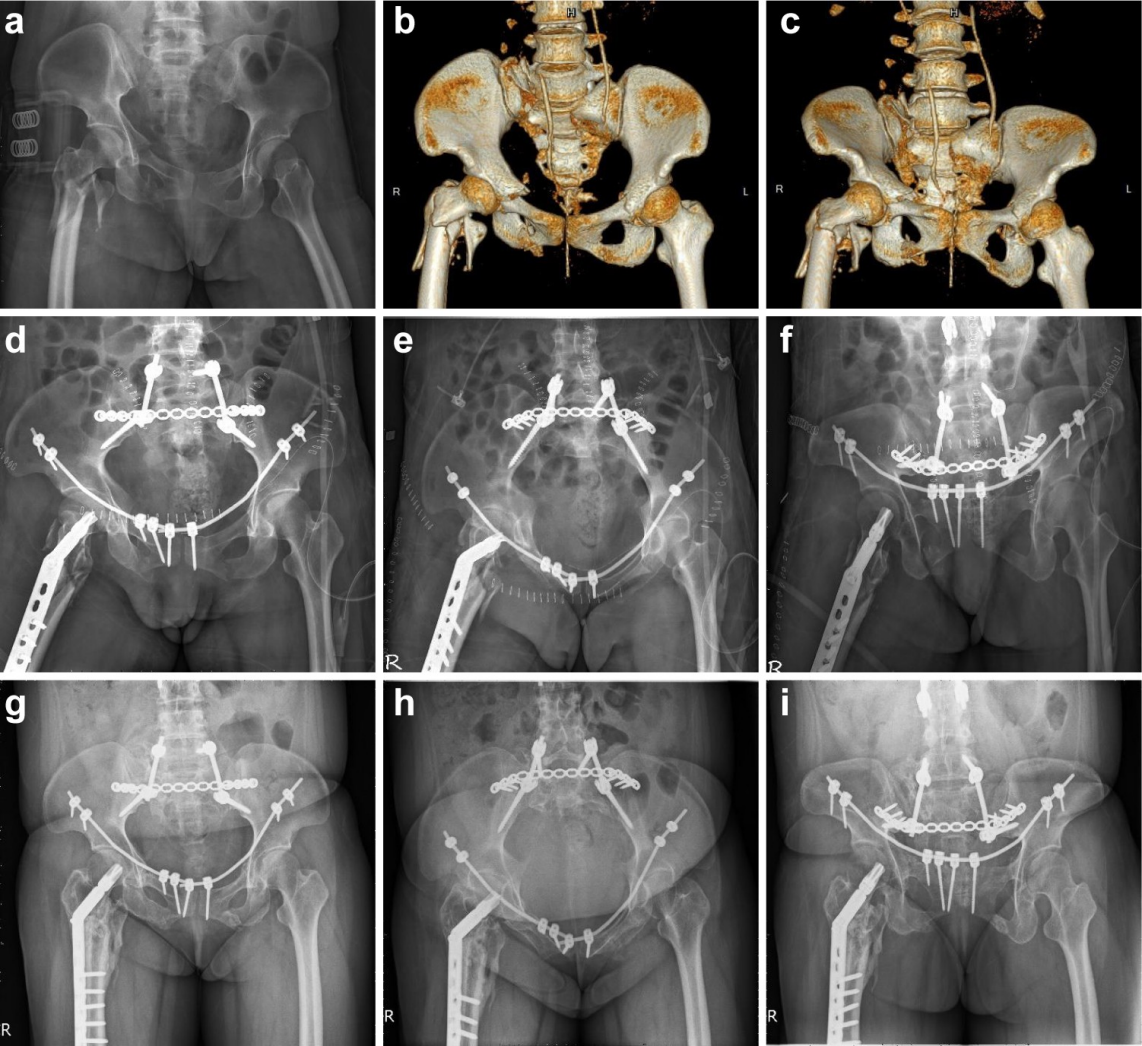
A 37-year-old female with anterior and posterior pelvic ring injury. **a** Preoperative pelvic anterior-posterior (AP) view. **b** Preoperative 3D reconstructed computed tomography (CT) AP view. **c** Preoperative 3D reconstructed CT outlet view showing a U-shape sacral fracture with bilateral superior and inferior pubic rami fractures and comminuted right proximal femur fracture. **d, e, f** Postoperative pelvic radiology series (AP, inlet, outlet view) demonstrating spinopelvic and transiliac plate fixation of the posterior pelvic ring fracture, bilateral MPSRF of the anterior pelvic ring fracture, and dynamic hip screw fixation of the right proximal femur. **g, h, i** Postoperative pelvic radiology series (AP, inlet, outlet view) at 13-month follow-up, demonstrating bone union of the pelvic ring. The quality of fracture reduction was graded as excellent.

In group 1, excellent, good, and fair grades were observed in 3, 3, and 1 cases, respectively (Fig 2). In group 2, excellent, good, and fair grades were observed in 6, 1, and 0 cases, respectively (Fig 3). There was no significance difference (P = 0.266) between the groups in the fracture reduction quality. Table 3 shows the clinical outcomes.

**Table 3:** Clinical outcomes.

	Group 1 (n = 7)	Group 2 (n = 7)	**P value**
**Operative time**	280.86±79.68	245.00±66.39	0.378^a^
**Blood loss**	601.43±153.78	337.14±229.11	0.026^a^
**Radiographic grading**			0.266^b^
** Excellent**	3	6	
** Good**	3	1	
** Fair**	1	0	
**Complications**			
** No**	5 (71.4%)	6 (85.7%)	
** Yes**	2 (28.6%)	1 (14.3%)	

n: number; M±SD: mean±standard deviation

^a^t test or chi-square test

^b^Fisher’s exact test

### Postoperative complications

Two patients developed complications in group 1. Plate loosening and loss of reduction were noted in one patient at 4 weeks post-surgery. Revision open reduction and internal fixation with plate surgery were performed in this patient. One patient sustained urinary bladder rupture with vesico-cutaneous fistula formation. In group 2, one patient sustained rod breakage 4 months after surgery, but complete fracture healing without discomfort was diagnosed. No further surgery was needed. No surgical wound infections were diagnosed perioperatively. No iatrogenic nerve injury (LFCN or femoral nerve palsy) occurred after surgery. No patient was diagnosed with nonunion or delayed union on any of the follow-up radiographic studies.

## Discussion

Successful pelvic fracture surgery remains a challenge for orthopedic surgeons due to the complex anatomy and fracture pattern. These patients also have a high percentage of associated injuries (primary hit). Thus, short surgical time and reduced blood loss in pelvic surgery are important factors to prevent secondary hit in these patients. Extensive surgical dissection to expose the pelvic brim is required in conventional surgery using plate fixation for anterior pelvic fracture repair. This may aggravate surrounding tissue damage and increase intraoperative blood loss. After the fracture site had been reduced, contouring the plate is time-consuming during the operation [11]. In our study, compared to conventional plate fixation, MPSRF had some advantages, especially in terms of reduced blood loss (with significant difference) and shorter operative time. Limited dissection of the iliacus muscle from the ilium could reduce blood loss with the MPSRF method. Compared to contouring the plate intraoperatively, bending the rod was easier and less time-consuming. A polyaxial pedicle screw was used in our study and can reduce the difficulty of rod application and manipulation, also reducing operative time. Meena et al. reported that the modified Stoppa approach provided a shorter operative time when compared to the ilioinguinal approach in anterior acetabular fracture [12]. In our study, different surgical approaches might have led to further reduction in operative time in group 2.

The use of pedicle screw-rod fixation as a minimally invasive method for anterior pelvic ring fixation had been developed in recent years. However, Vigdorchik et al. concluded that plate fixation for anterior pelvic ring fracture had higher stability than the INFIX and external fixator in a biomechanical study [13]. In one review paper, INFIX and appropriate posterior fixation resulted in healing of pelvic ring injuries in 99.5% of cases [14]. In our study, we did not perform a biomechanical study to compare the fixation stability of the fracture site between the modified pedicle screw-rod fixation and the neutralization plate. However, bone union was noted in all patients. This could be because most of the patients in the MPSRF group received stable fixation for the posterior ring (such as spinopelvic fixation and transiliac plate fixation). Seventy percent of the stability of the pelvic ring was provided by the posterior pelvic ring. When the patient achieved stable posterior fixation, the anterior pelvic ring could be fixed with the MPSRF. Postoperative rehabilitation protocol plays an important role. In this study, non-weight bearing of the injured limb for 6 weeks and partial weight bearing was allowed when partial callus formation was observed in the follow-up radiography. Too early weight bearing may cause loss of reduction of the pelvic ring. The iliac fossa region was very thin for screw purchase. To prevent unsecured screw fixation, we modified the pedicle screw position approximately 4–5 cm medial to the ASIS. The iliac bone was thicker in this region. In our study, the screw length used in the iliac wing region was approximately 14–18 mm. The inner cortex and outer cortex of the iliac bone could be firm purchased by the pedicle screw. If the screw fixation was unsecured during the surgery, we changed the screw to an appropriate position. Moreover, the 4.0 mm-diameter cervical pedicle screw used in the iliac wing fixation was larger than the 3.5-mm diameter cortical screw used for the conventional reconstruction plate. In our study, no screw loosening complication was observed. One patient sustained rod breakage, but no discomfort or symptoms were reported by the patient. Thus, this MPSRF technique provided appropriate stability for clinical fixation of anterior pelvic ring fracture.

Complications of INFIX include LFCN irritation, heterotopic ossification, infection, femoral nerve palsy, and painful implant impingement [14,15]. In a cadaveric study, the femoral nerve was the structure at most risk of compression by the INFIX rod [16]. In our MPSRF technique, we used a small-diameter pedicle screw (3.5-mm and 4.0-mm) rather than the large pedicle screw (6.5-mm or 7.5-mm) used in INFIX, and we changed the pedicle screw insertion site from the anterior inferior iliac spine to the inner table of the ilium, near the ASIS. No cases of LFCN injury or impingement by the pedicle screw were noted. The connecting rod used in the MPSRF technique was applied under the muscular and neurovascular layers. This could minimize the risk of femoral nerve injury by preventing the occurrence of a rod compression force. The axis of pedicle screw insertion was an important factor to reduce patient discomfort due to soft tissue irritation and urinary bladder damage. The ideal axis is perpendicular to the superior surface of the superior pubic rami, allowing the pedicle screw head to be covered by abdominal muscles, making it non-palpable and non-irritable. In addition, the axis from the anterosuperior to posteroinferior regions may elevate the risk of urinary bladder injury. The vertical axis can prevent the sharp tip of the pedicle screw from injuring the urinary bladder.

Approximately 15% of patients with pelvic fractures have associated bladder or urethral injuries [17]. In our study, one patient in group 1 sustained extraperitoneal urinary bladder rupture without secure repair during the pelvic surgery. Vesico-cutaneous fistula formation developed as a late complication. Bladder repair is limited with the ilioinguinal approach. Another advantage of the modified Stoppa approach, used in the MPSRF group, was an intra-pelvic approach, which could more easily repair extraperitoneal bladder rupture.

There are some limitations of our investigation, including the small case number, retrospective design, and absence of a biomechanical study. A larger patient population is needed to further assess the clinical application of our technique. A randomized, controlled, blinded study evaluating long-term clinical outcomes may establish more evidence-based conclusions. In addition, a biomechanical study could provide more convincing result. Further studies should include a prospective analysis to evaluate the effect of the MPSRF method on treatment outcomes.

Overall, this modified pedicle screw-rod fixation technique is a reliable treatment for anterior pelvic ring fracture. Most complications associated with INFIX could be prevented by this modification. When compared with conventional plate fixation methods, this new technique could reduce surgical time and intraoperative blood loss.
